# The bumpy road to implementing the Baby-Friendly Hospital Initiative in Austria: a qualitative study

**DOI:** 10.1186/s13006-015-0030-0

**Published:** 2015-01-20

**Authors:** Christina C Wieczorek, Hermann Schmied, Thomas E Dorner, Wolfgang Dür

**Affiliations:** Ludwig Boltzmann Institute Health Promotion Research, Ludwig Boltzmann Gesellschaft, Untere Donaustraße 47, 1020 Vienna, Austria; Institute of Social Medicine, Centre for Public Health, Medical University of Vienna, Kinderspitalgasse 15, 1090 Vienna, Austria

**Keywords:** Baby-Friendly Hospital Initiative, Breastfeeding, Health services research, Diffusion of innovation

## Abstract

**Background:**

The Baby-Friendly Hospital Initiative (BFHI) aims to promote and support breastfeeding. Globally, around 20,000 facilities have been designated Baby-Friendly. In Austria, however, only 16% of the maternity units have received BFHI-certification. Internationally, few studies have investigated facilitating or hindering factors for BFHI implementation. The need to extend BFHI-certification rates has been investigated previously, but little is known about *why* maternity units decide to become BFHI-certified, *how* BFHI is installed at the unit level, and *which factors* facilitate or impede the operation of the BFHI in Austria and how barriers are overcome.

**Methods:**

Using a qualitative approach, (health) professionals’ perceptions of the selection, installation, as well as facilitators of and barriers to the BFHI were investigated. 36 semi-structured interviews with persons responsible for BFHI implementation (midwives, nurses, physicians, quality manager) were conducted in three Austrian maternity units. Data were analyzed using thematic analysis.

**Results:**

Interviewees mentioned several motives for selecting the BFHI, including BFHI as a marketing tool, improvement of existing services, as well as collaboration between different professional groups. In each hospital, “change agents” were identified, who promoted the BFHI, teamed up with the managers of other professional groups and finally, with the manager of the unit. Installation of BFHI involved the adoption of project management, development and dissemination of new standards, and training of all staff. Although multiple activities were planned to prepare for actually putting the BFHI into practice, participants mentioned not only facilitating, but also several hindering factors. Interpretations of what facilitated or impeded the operation of BFHI differed among and between professional groups.

**Conclusion:**

Successful implementation of the BFHI in Austria depends on a complex interplay of multiple factors including a consensual “bottom-up” selection process, followed by a multifaceted installation stage. Even these activities may be perceived as a hindrance for non-BFHI-certified hospitals. Findings also suggest that despite active preparation, several barriers have to be overcome when BFHI is actually incorporated into routine practices. BFHI seems to pose a great challenge to health professionals’ work routines and, thus, clear structural changes of such routines as well as ongoing monitoring and support activities are required.

## Background

The advantages of breastfeeding for infants and their mothers are well known [1,2]. To protect and promote breastfeeding globally, the World Health Organization (WHO) and the United Nations Children’s Fund (UNICEF) launched the Baby-Friendly Hospital Initiative (BFHI) in 1991. BFHI aims to promote and support breastfeeding in environments, such as maternity units, and recommends at least six months of exclusive breastfeeding and continued breastfeeding for two years or beyond. BFHI includes the Ten Steps to Successful Breastfeeding (hereafter referred to as the Ten Steps) (see Table 1) that must be fulfilled by a hospital/maternity unit to become a “Baby-Friendly” hospital [3].

**Table 1:** **Ten steps to successful breastfeeding [** 3 **]**

**Every facility providing maternity services and care for newborn infants should:**	
1.	Have a written breastfeeding policy that is routinely communicated to all health care staff.
2.	Train all health care staff in skills necessary to implement this policy.
3.	Inform all pregnant women about the benefits and management of breastfeeding.
4.	Help mothers initiate breastfeeding within one half-hour of birth.^*^
5.	Show mothers how to breastfeed and maintain lactation, even if they should be separated from their infants.
6.	Give newborn infants no food or drink other than breast-milk, unless medically indicated.
7.	Practice rooming in – that is, allow mothers and infants to remain together 24 hours a day.
8.	Encourage breastfeeding on demand.
9.	Give no artificial teats or pacifiers (also called dummies or soothers) to breastfeeding infants.
10.	Foster the establishment of breastfeeding support groups and refer mothers to them on discharge from the hospital or clinic.

^*^Since 2009, this Step is interpreted as: “Place babies in skin-to-skin contact with their mothers immediately following birth for at least an hour. Encourage mothers to recognize when their babies are ready to breastfeed and offer help if needed” [4].

In addition to the Ten Steps, the International Code of Marketing of Breast-Milk Substitutes (in the following referred to as the Code) has to be observed and, since 2009, depending on national decisions the criteria for mother-friendly care must also be included [3,4]. Currently about 20,000 hospitals in more than 150 countries have ever been designated Baby-Friendly [5]. Multiple studies show that the implementation of BFHI increases the initiation and, less strongly, the duration rates of any and exclusive breastfeeding [6-9].

In Austria, as in many other European countries, initiation of breastfeeding is high [10]. 93.2% of mothers initiate breastfeeding, yet the latest available statistics indicate that the rates of exclusive breastfeeding have dropped to 9.7% by six months [11]. This data shows that only a minority of infants in Austria are breastfed according to the recommendations of WHO and UNICEF. The data also supports international data that mothers in BFHI-certified facilities are more likely to initiate and continue breastfeeding [6-9,11,12]. However, to date, only 16% of the maternity units in Austria have been designated as Baby-Friendly [13], although the BFHI was launched in this country in 1996. Compared to some other countries, especially in northern Europe, BFHI-certification rates are rather low [14]. These findings, as well as others that will be outlined below, make the Austrian case an interesting one.

Generally, there is a mandatory statutory insurance system and hospital care is publicly funded in Austria. Nationwide, there are 79 maternity units, from which insured women can make a selection. Hospital owners are mainly public authorities, such as municipalities, provinces, and social insurance companies or private bodies, such as religious orders. In addition, there are a few private, for-profit hospitals where women can give birth at their own expense. In 2012, there were 78,952 births in Austria. In the last ten years, this number of births has been quite constant [15]. By contrast, cesarean section rates have increased massively, by about 43% between 2002 and 2012, as they have in most developed countries [15]. In Austria, the cesarean section rate was 28% in 2011 and thus slightly above the OECD average of 27% [16].

While in the early years, UNICEF Austria supported the BFHI and BFHI-certification was carried out by a few individuals, since 2010, it has been coordinated and carried out by a specialized BFHI division of the Austrian Health Promoting Hospitals Network. This division also determined that the criteria for mother-friendly care have to be fulfilled by Austrian maternity units to gain BFHI-certification (see Table 2). Moreover, between 2011 and 2013, BFHI was supported financially by the Austrian Federation. Finally, as part of the financial support, BFHI has been promoted through multiple roll-out actions.

**Table 2:** **Criteria for mother-friendly care in Austria [** 13 **]**

**A natural birth experience is a significant prerequisite for successful breastfeeding. Therefore, mother-friendly care is a compulsory part of BFHI-certification. The criteria require, unless medically indicated that:**	
a)	Mothers can bring a companion of their choice to provide continuous physical and/or emotional support during labor and birth, as desired.
b)	Mothers can drink and eat light foods during labor, as desired.
c)	Mothers can walk and move about during labor, as desired.
d)	Mothers can choose a position while giving birth.
e)	Mothers should be offered the use of non-drug methods of pain relief.
f)	Invasive procedures such as rupture of the membranes, episiotomies, acceleration or induction of labor, instrumental deliveries, or cesarean sections should be used only for medical indications.
g)	Standards, guidelines and training curricula of the maternity unit support mother-friendly care.

The aim of our study was to investigate why BFHI-certification rates in Austria are rather low, compared to some other countries, and what factors or barriers have considerable influence on the implementation of BFHI in Austrian hospitals. A needs assessment has shown that experts generally recognize the need for BFHI and emphasize expansion of BFHI-certification rates in Austria [17]. Internationally, a few studies have investigated facilitators of and barriers to BFHI implementation [18-20] as well as health professionals’ perceptions of BFHI implementation [21-24]. A systematic review by Greenhalgh et al. [25] identified several determinants of change in health service delivery and organizations. Logical models of program implementation in organizations complement this review by specifying chronological stages of the implementation process [26,27]. Following these models, we differentiate three main stages of implementation for the purpose of our study:

1. *Selection*: At some point, individuals or organizations become aware of an evidence-based program. The purpose of this stage is to assess the match between the program and existing individual and organizational needs as well as resources. At the end of this stage, a decision is made on whether to proceed or not.
2. *Installation*: After the decision is made to implement the particular program, several activities, such as the set-up of supporting structures or additional resources need to be completed before the program is put into practice.
3. *Operation of the program*: Finally, new program-related practices are incorporated into the routine practices of the organization.

Following these stages of implementation, our paper aims to illuminate how health professionals in Austria perceived (1) the *selection of BFHI*, (2) the *installation of BFHI* at the unit-level, and (3) *facilitators of and barriers to BFHI operation*. We further pursue the question of how the facilities have overcome the different barriers.

## Methods

### Study design

In this study, a qualitative approach [28], including individual, semi-structured interviews and “thematic analysis” [28] was chosen as the method for interpreting the data. This design was considered the most suitable data collection method because it allowed for investigating perceptions of different professional groups (midwives, nurses, physicians, and quality manager) of the three stages of BFHI implementation.

### Study setting

The study took place in three hospitals/maternity units in an urban area of Austria. Of these, two were publicly owned and one privately (by a religious order), but each unit had public access for women with the obligatory health insurance. The three maternity units together had around 6,000 births per year and nearly all mothers were in-patients. Only 254 (4.3%) had out-patient births in 2012, i.e. mothers gave birth in the hospital and were discharged after a few hours as there were no complications. Further supervision was provided by a midwife and a pediatrician at home. Two hospitals had been BFHI-certified during the last two years and one hospital was preparing for its first BFHI-certification at the time our study took place. According to the BFHI division of the Austrian Health Promoting Hospitals Network, since 2010, BFHI-recertification would be demanded after four years.

### Participant selection

Participants were recruited using purposive sampling [28] to ensure that interviewees reflected a wide variety of perspectives on the selection, installation, and both the facilitators of and barriers to BFHI operation. In each hospital we aimed to recruit: a) Participants from all professional groups (midwives, nurses, pediatricians, gynecologists, and anesthesiologists) and b) Staff in a management position as well as staff working in a non-executive position. In particular, the number and composition of participants was arranged with each maternity unit. The managers of each professional group were asked to participate and to inform and nominate at least two colleagues, preferably with different years of work experience. Each designated participant was phoned individually by the lead author to give further information about the study, to agree upon the participation and if so, to arrange a date and time for the interview. To minimize recruitment pressure among employees in a subordinate position, only those who were available and willing to participate in the study were nominated and recruited. A total of 35 health professionals (11 nurses, 11 midwives, 13 physicians) working across the three maternity units and one quality manager who was involved in the installation of BFHI, participated in the study. Table 3 provides further details about the participants.

**Table 3:** **Overview – participants’ profile**

**Participant and occupation**	**Gender**		**Position**		**Years of work experience**		
	**Female**	**Male**	**Management**	**Non-executive**	**<5 years**	**5 – 15 years**	**>15 years**
Hospital A* (n = 14)							
Physicians (n = 5)	5		1	4	1	2	2
Midwives (n = 5)	5		1	4		2	3
Nurses (n = 4)	4		1	3		1	3
Hospital B* (n = 11)							
Physicians (n = 4)	3	1	1	3		2	2
Midwives (n = 3)	3		1	2	1		2
Nurses (n = 4)	4		2	2		1	3
**Hospital C (n = 11)**							
Physicians (n = 4)	3	1	1	3		2	2
Midwives (n = 3)	3		1	2	1		2
Nurses (n = 3)	3		1	2		3	
Quality manager (n = 1)		1	1			1	

^*^Already BFHI-certified.

### Data collection

Data were collected between August and December 2013 with two researchers being present at each interview. Each interview was held in the hospital in which the interviewee worked and lasted approximately one hour. The main themes of the interview guide followed the logical models of program implementation in organizations [26,27]. An overview of the questions used is given in Table 4. All interviews, except one where we did not receive permission, were digitally recorded and transcribed verbatim. For the one exception, one of the interviewers took field notes.

**Table 4:** **Key prompts – individual, semi-structured interviews**

**Personal information**	Please describe your position and field of action/responsibilities in this hospital.
	What is your role in relation to the implementation of the BFHI?
**Selection of BFHI**	Why do you think BFHI was selected?
	Who was involved and how was it decided to become Baby-Friendly?
**Installation of BFHI**	Once the decision to become Baby-Friendly has been made, what are the next steps to prepare BFHI operation?
**Facilitators of and barriers to BFHI operation**	Please describe the operation of the BFHI.
	What are the challenges that your hospital experienced in becoming Baby-Friendly?
	What are your general views and opinions about the BFHI?
	How were barriers overcome?

### Data analysis

Data were analyzed using thematic analysis [28]. This was a repetitive process which involved identifying and labeling codes following the main themes of our interview guide, as well as themes which appeared in the data. Subsequently, relevant categories and characteristics were defined for each theme. In the end, summaries for each theme were generated and linkages between codes were investigated to answer the research questions. All steps were carried out using the research software Atlas.ti, developed for qualitative data analysis. To enhance the rigor of data analysis, CCW and HS each coded some of the transcripts once a coding scheme had been developed by the research group. Moreover, ongoing discussions on key findings were undertaken with TED and WD. Summaries and field notes were used to document the process. Quotes were used to enhance the credibility of the findings. To ensure confidentiality, all participants were assigned pseudonyms, only indicating their professional group and number, e.g. Midwife 8.

### Ethical approval

Ethical approval was obtained from the relevant Ethics Committee prior to the commencement of the study (EK 13-188-VK_NZ). All participants were provided with written information about the content and the purpose of the study. As noted above, except for one participant, all gave written consent to record the data. The participants’ confidentiality and autonomy were protected at all stages.

## Results

The results are presented in accordance with the themes investigated: (1) Selection of BFHI, (2) Installation of BFHI, and (3) Facilitators of and barriers to BFHI operation. Concerning the barriers to BFHI operation, also data on how maternity units overcame each barrier will be emphasized. While most themes were consistent across all three sites, a few only emerged for one or two hospitals, but appeared to be important. These will be highlighted in the text.

### Selection of BFHI

#### Motives

On the whole, interviewees of all sites mentioned multiple motives for becoming a Baby-Friendly hospital, ranging from BFHI-certification as a marketing tool, to improvement of existing services, and improvement of collaboration among professional groups (see Table 5). No considerable differences between the professional groups or between staff in a management position versus staff in a non-executive position could be found.

**Table 5:** **Results of the selection of BFHI – overview of categories and sub-categories**

**Category**	**Sub-category**
Motives	Marketing tool
	Improvement of existing services
	Improvement of collaboration among professional groups
Promoters and decision-making	Individual persons in the role of change agents
	Consensus of managers of different professional groups
	Consent of top management

##### Marketing tool

Most participants viewed the BFHI as a marketing tool for the hospital. They described the BFHI as a means to gain more publicity, to attract mothers and to set the unit apart from other maternity units. One midwife pointed out: “Certification is an accentuating attribute. You set yourself apart from other hospitals” (Midwife 8). Likewise, other respondents emphasized that BFHI-certification was a “calling card” (Nurse 6) for the hospital as there are only a few designated maternity units in Austria. They explained that BFHI was particularly important for a positive corporate image of a maternity unit.

Several participants also noted that BFHI-certification was a credible quality label and would, therefore, support maternity units in fulfilling mothers’ or parents’ expectations of high-quality service delivery on maternity units.

##### Improvement of existing services

Another motive to become Baby-Friendly was the improvement of existing services and work procedures on maternity units. Typically, the improvement of existing services referred to aspects such as encouraging the close relationship between mother and baby as well as not separating mother and baby, but also involved a paradigm shift. This paradigm shift was described as moving from having the staff rather than the mother care for the baby to empowering the mother to care for her baby herself. As a pediatrician said: “[…] from an active person who cares for the baby to a coach for the mother” (Physician 6). Working according to the BFHI criteria was seen as an enhanced form of supporting and caring for mothers and their babies.

##### Improvement of collaboration among professional groups

A number of participants also noted that BFHI would be of benefit for interdisciplinary collaboration on the unit. They emphasized that on maternity units, multiple professional groups, such as nurses, physicians, including gynecologists, pediatricians and anesthesiologists, but also midwives, had to interact in multiple working processes. Yet, interviewees felt that there was often room for improvement in inter-professional collaboration. Through BFHI, participants expected to achieve an “interdisciplinary convergence” (Quality manager 1). Interviewees indicated that BFHI was not just expected to improve existing services on the unit, but also to change health professionals’ practices and patterns of collaboration.

#### Promoters and decision-making

Apart from motives, all participants were asked to describe who promoted the BFHI on their unit and how the decision to become Baby-Friendly came about (see Table 5).

##### Individual persons in the role of change agents

Most respondents emphasized that just a few colleagues promoted the program at the beginning. We call them “change agents”. These change agents were individual persons, (directly) located on the maternity unit. Analysis revealed that change agents were personally convinced about the content and advantages of the BFHI as such, but also about the benefits of BFHI-certification for the hospital. Another characteristic of the change agents was that they were also able to influence the managers of the other relevant professional groups. In all cases, the nurse unit manager was among the change agents. One nurse unit manager pointed out: “[…] I, myself, promoted this project. I’ve really been the engine to push it forward and to get the managers of the other professional groups on board” (Nurse 9). Participants emphasized the importance of the presence of some change agents to facilitate the move from advocating the BFHI to an organizational decision to become BFHI-certified.

##### Consensus of managers of different professional groups

To decide upon seeking BFHI-certification, participants typically referred to the relevance of the managers of different professional groups. These included the manager of midwives, the nurse unit manager, the manager of pediatricians, as well the manager of the whole maternity unit, who was, in all hospitals, a gynecologist. Analysis showed that consensus among all managers of the professional groups as well as the manager of the unit was required to move towards a decision about whether to become Baby-Friendly. In one hospital, the manager of the unit explained that, during a weekend meeting with all relevant managers, basic points about the BFHI had been agreed upon. In the other two hospitals, participants said that an agreement to become a Baby-Friendly hospital was made during a regular team meeting. The manager of one of these units clarified: “[…] we have a strategic meeting with all managers of the professional groups and there we decided to do it [become BFHI-certified].” (Physician 3)

##### Consent of top management

For the formal decision to pursue BFHI-certification, the consent of the hospitals’ top management was characteristically required. The quality manager noted: “[…] the order is given by top management. […] yet, the input often comes from the bottom.” (Quality manager 1) In one hospital, some interviewees emphasized that, for long time, it was only the lack of consent by one member of the top management that prevented the decision to earn BFHI-certification from being made.

### Installation of BFHI

All interview participants were asked which steps were taken next to prepare for and support the operation of the BFHI in the next stage. Data analysis showed (a) project management, (b) development and dissemination of new standards, and (c) training of all health care staff (see Table 6).

**Table 6:** **Results of the installation of BFHI – overview of categories and sub-categories**

**Category**	**Sub-category**
Project management	Launch of a project group
	Launch of sub-groups
Development and dissemination of new standards	Manifestation of new work procedures
	Dissemination of Baby-Friendly standards
Training for all health care staff	Participation in training following Austrian training requirements
	Training as a lactation consultant

#### Project management

##### Launch of a project group

Interviewees in all hospitals said that a project group was established, which was responsible for the overall BFHI-certification process as recommended by the Austrian BFHI-certification body. Typically, the managers of the professional groups as well as employees in a non-executive position built the project group. Non-executive project group members were those, who were convinced about the substance and benefits of the BFHI. The quality manager noted further: “The interdisciplinary project group consists of all persons who are responsible for such a project” (Quality manager 1). The project group met at least once a month over a period of one to two years.

To facilitate project group meetings, participants said that top management and the managers of the professional specialties had agreed that meetings could take place during work time. Yet, a number of participants felt that meetings were rather intensive and time-consuming and dedicated work time was insufficient: “[…] it’s the meetings as such, and sitting together, and preparing these meetings which require a lot of time and effort” (Physician 13).

##### Launch of sub-groups

In addition to the project group, several sub-groups were created to work on particular aspects/themes required by the BFHI. A nurse in the hospital preparing for its first BFHI-certification explained: “We’ve a sub-group which works on breastfeeding, a group working on breastfeeding statistics, a group working on training and workshops, a group working on bonding [skin-to-skin contact] […]” (Nurse 9). By comparison, the other two hospitals had fewer sub-groups. Here, sub-groups elaborated on multiple aspects of the BFHI.

As with the project group, meetings of sub-groups could take place during work time. Considering the composition of sub-groups, most interviewees emphasized that it depended on the particular topic. However, several highlighted the fact that representation of all relevant professional groups was ensured. Participants in a management position further noted that the purpose of the multiple sub-groups was not merely the elaboration of new work procedures manifested in the new standards, but that sub-groups were also thought to be a means to gain staff commitment on the unit. This was expected to facilitate BFHI operation later on. However, this part of the agenda was enacted differently by the three hospitals. While two hospitals mainly searched for fellow travelers, staff members who were convinced about the content and benefits of the BFHI, one hospital deliberately addressed resisters: “We also try to involve the so-called detractors in our sub-groups and thus to let them participate in the preparation of the project” (Physician 11).

#### Development and dissemination of new standards

##### Manifestation of new work procedures

A main task of the sub-groups was the development of standards which included a description of new BFHI-related work procedures as well as distribution of responsibilities. Besides the required written breastfeeding policy (Step 1) a couple of additional standards, e.g. standardizing skin-to-skin contact after cesarean sections or documentation forms for breastfeeding statistics, were prepared. Interviewees repeatedly emphasized that the development of the new standards depended on inter-professional agreement. This was considered a basic prerequisite for successfully putting BFHI standards into practice for two reasons. First, inter-professional agreement on new standards was thought to prevent professionals from feeling disregarded. Second, it was expected that this strategy of developing new standards would reduce the risk of failure once the standard became adopted in daily routines: “We’ve tried to learn from the failures of other hospitals, which had forgotten about particular professional groups. I really tried to include everyone. For example, having a young anesthesiologist […] on board would be helpful, especially to inform and get commitment from his colleagues” (Nurse 9).

##### Dissemination of Baby-Friendly standards

Once new standards were defined, these standards had to be conirmed by the manager of the maternity unit. To facilitate the dissemination of the new standards, interviewees referred to the usage of multiple sources. In particular, dissemination took place during intra-professional team meetings: “First of all, it was discussed in every team meeting. It [the BFHI] was a key issue in every team meeting” (Nurse 8). Employees were also informed face-to-face by members of the sub-groups or project group and standards were propagated in written form (e.g. via mail). Most participants felt that multiple dissemination sources helped to ensure that every employee working on the maternity unit recognized and became familiar with new work procedures. Interviewees further explained that after dissemination of new standards, every employee was expected to work according to those standards from that time onwards: “[…] innovations are spread via e-mail and every employee is expected to read it [the e-mail] because from that time onwards working according to the innovation is compulsory” (Midwife 8).

#### Training of all health care staff

Step 2 of the international BFHI-certification requirements requires mandatory training for health care staff to implement BFHI.

##### Participation in training following Austrian training requirements

The Austrian BFHI certification authority has defined the scope of training as 20 hours for midwives and nurses, 10 hours for physicians and four hours for nursing assistants working in the maternity unit. Therefore, the hospitals implemented comprehensive internal and external training programs. A nurse explained it as follows: “We had a schedule for all trainings. For all employees we had a two week training which was quite intensive […]” (Nurse 5).

To contain costs, participants of all sites emphasized, as did this nurse, that internal resources were used. Moreover, to keep costs low, experts from other hospitals, already BFHI-certified, were invited to give an “in-house” training/workshop: “A lot of training was provided on site by persons who were familiar with the BFHI. However, we also invited people from other hospitals [already BFHI-certified] to share their experience with our staff” (Nurse 2). Interviewees noted that internal training was cheaper than sending all staff to external training. However, data showed that midwives and nurses often fulfilled their training requirement by participating in the basic course offered by the European Institute for Breastfeeding and Lactation (EISL).

##### Training as a lactation consultant

Some staff also took the training towards the International Board Certified Lactation Consultant (IBCLC) examination. Although the training towards IBCLC examination goes far beyond the Austrian training requirements, most participants mentioned the importance of this training. They felt that this training could facilitate BFHI because IBCLC-certified staff had expert knowledge on lactation and lactation problems.

All three hospitals supported staff in gaining IBCLC-certification by covering costs. Cost coverage was possible as long as IBCLC-training was considered within the strategic planning of the hospital. However, some participants commented that cost coverage was not endless and pointed out: “In the beginning, costs were partly refunded but some day, of course, management said that we’ve already done that much… it’s enough” (Nurse 8).

### Facilitators of and barriers to BFHI operation

Thematic analysis of the data has resulted in several facilitators of and barriers to putting BFHI into practice (see Table 7).

**Table 7:** **Results of the facilitators of and barriers to BFHI operation – overview of categories and sub-categories**

**Category**	**Sub-category**
Facilitators of BFHI operation	Skills of the staff
	Management support
	Getting staff on board
Barriers to BFHI operation	Lack of time and staff resources
	Old patterns
	Personal experiences
	Lack of physician buy-in
	Tensions between care for mothers and care for babies
	Intra- and inter-professional discontinuation of the BFHI care-chain
	Language and literacy barriers of mothers and their relatives
	Expectations of mothers and their relatives

#### Facilitators of BFHI operation

##### Skills of the staff

There was a common perception that the required training program for all staff was an important measure to ensure necessary skills and competences for executing the BFHI. Typically, the training towards IBCLC examination was discussed when highlighting skills of staff facilitating BFHI. Participants explained that IBCLC-certified staff included midwives, nurses, and also some physicians and this staff facilitated the BFHI operation because they were internal experts who shared their knowledge with colleagues and supported them: “[…] we can always ask for advice and they [IBCLCs] keep us updated.” (Nurse 1) Another function of IBCLC-certified staff was the care for mothers with breastfeeding problems. One nurse said: “We particularly pay attention that IBCLC-nurses take it [mothers with breastfeeding problems] over from non-IBCLC-certified employees” (Nurse 7). Some IBCLC-nurses commented critically that supporting colleagues and taking care of mothers with breastfeeding problems often meant an additional work load for themselves because they had no dedicated working time for this assistance. In one hospital, IBCLC-nurses were relieved from regular duties on the unit and were appointed to concentrate on supporting colleagues and mothers on lactation.

##### Management support

Most interviewees assessed support by the managers of all professional groups, as well as the manager of the maternity unit, as facilitating the BFHI. The quality manager noted: “It’s the manager of the unit, the nurse unit manager, the manager of midwives who should or even have to facilitate the project […]” (Quality manager 1).

Some interviewees felt that the managers of each professional group were required to convince their colleagues. A nurse pointed out: “Support from the manager of the unit. He has to stand behind us. Especially in case some physicians oppose the program, he’s needed to reprimand these physicians” (Nurse 6). A pediatrician supported this by providing a reverse example. This person explained that on the maternity unit, the manager of midwives had not given her support and consequently putting the BFHI into practice had been challenging, if not almost impossible in the past.

##### Getting staff on board

Almost all referred to the relevance of a commitment to facilitate BFHI during daily routines and emphasized that this was an ongoing process. Interviewees stressed that a lot of time and effort was required to get a critical mass of staff members on board. Interviewees emphasized that to put BFHI into practice on the unit level, over hundred staff members had to be involved. Resistance and concerns of staff should be minimized through multifaceted persuasive efforts. A nurse remembered: “It took a long time to convince me that it’s [BFHI] worthwhile. […] Yet in the end I was even involved in committing other colleagues” (Nurse 8). Many interviewees felt that achieving commitment and acceptance from staff was a “bumpy road” (Nurse 6).

To achieve commitment, all hospitals conducted activities, in addition to those set during the installation stage. It was explained that presenting the evidence of health benefits through breastfeeding to staff, showing positive outcomes of Baby-Friendly practices already implemented, e.g. by breastfeeding statistics, as well as ongoing discussions during team-meetings, helped to increase commitment. Analysis also showed that two hospitals had a kick-off meeting in the beginning. One nurse unit manager pointed out: “It started with a huge kick-off meeting for the whole hospital. It’s really important, really important that all know what you’re doing. Everybody needs to be informed […]” (Nurse 5).

#### Barriers to BFHI operation

##### Lack of time and staff resources

Several participants felt that working according to BFHI standards needed more work time in particular situations. Often they either referred to the intensive support of breastfeeding and, thus, the increased communication efforts in daily work with mothers, or to skin-to-skin contact after cesarean section. With respect to skin-to-skin contact after cesarean section, participants pointed out that midwives had to stay in the theatre for a while. In the past, the midwife could return directly to the labor room or maternity unit and fulfill other tasks. A nurse unit manager noted particularly: “It’s not that easy. In times of staff shortages, no one wants to carry additional duties. That’s really challenging” (Nurse 9). Thus, required working time and increased workloads were seen critically, not least in combination with staff shortages. As a response, the two hospitals already BFHI-certified had tried to rearrange work procedures and responsibilities to enable staff to fulfill BFHI standards.

##### Old patterns

Many participants saw persisting in old patterns as a major factor leading to resistance against the required changes. At all sites, this type of resistance rose with the number of years a staff member had worked on the maternity unit, especially among midwives and nurses. A midwife explained: “I’ve worked like that the past 25 years and I want continue doing so […]” (Midwife 2). A nurse unit manager similarly explained that older colleagues were more resistant because they considered the BFHI to be unnecessary “new rubbish” (Nurse 3). Several older midwives explained that they were used to bathing the baby directly after birth and to rapidly filling out the birth certificate. Yet, as a consequence of Step 4, writing the birth certificate could only be finished much later and as one midwife said “It [skin-to-skin contact] really disturbs established sequences” (Midwife 2). A gynecologist supported the relevance of old patterns and confirmed that younger midwives were more likely to adopt BFHI practices because they were familiar with these practices due to their recent education. Moreover, young midwives were thought not to have developed particular work patterns that would impede BFHI.

Others had the impression that former work practices were inadequate. Therefore, the acceptance of the BFHI standards would generate cognitive dissonance. A nurse unit manager explained: “[Colleagues] are concerned that care they’ve delivered so far isn’t ideal anymore […] and everything they’ve done in the past has been wrong or poorly carried out” (Nurse 3). Another nurse unit manager mentioned that one of her colleagues felt very guilty once she had finished BFHI training. Following the input from the training, this colleague felt that she must have done everything wrong in the past, even with respect to her own child.

To overcome old patterns, interviewees mentioned that, especially in the beginning, BFHI-related activities were discussed during regular team meetings. Some change agents referred to the importance of reassuring especially older colleagues, that they have probably always worked on the basis of the most up-to-date information available and that the BFHI merely adds some new insights. Other activities, to facilitate operation of the BFHI included face-to-face conversations among individual employees as well as obtaining feedback from mothers for whom BFHI-related practices had worked out well: “[…] and of course we’ve tried to gain feedback from mothers. Those where it [the BFHI] really worked out” (Physician 6). Finally, letting employees experience for themselves the success of BFHI was considered facilitating.

##### Personal experiences

Many participants commented that personal experiences of some staff hindered the BFHI operation, especially their personal experiences with breastfeeding or formula feeding. A nurse unit manager explained that she gave formula to her own child in the beginning and later started breastfeeding. She emphasized that breastfeeding worked quite normally. Others scrutinized BFHI and mentioned: “[…] I’ve not breastfed my own children and still, I’ve amazing kids” (Nurse 8). These participants explained that they felt some tension between their own experiences and what was required by the BFHI.

To overcome this barrier, convinced staff tried to discuss successful cases during team meetings. A lot of communication with resistant staff and trying to commit employees helped to reduce personal experiences as a hindrance for BFHI operation.

##### Lack of physician buy-in

Another considerable barrier was the lack of physician buy-in. This was particularly the case among anesthesiologists and gynecologists. The physicians themselves, as well as nurses and midwives, indicated that physicians were more likely to resist or at least were indifferent with respect to the BFHI. A nurse unit manager explained: “Especially among gynecologists […] there were some who refused it [BFHI]” (Nurse 9). A pediatrician spoke about the indifferent position of some colleagues and pointed out that their perception of responsibility was mixed.

In contrast to physicians, midwives and nurses already considered themselves as being responsible for BFHI operation. A midwife commented: “[BFHI] particularly applies to and involves midwives and nurses because they most directly interact with the women” (Midwife 2).

Analysis in the BFHI-certified hospitals showed that to facilitate BFHI, midwives and nurses advised physicians to refer to IBCLC-certified staff rather than giving wrong or contradictory responses to mothers with breastfeeding problems during their unit rounds. Nevertheless, midwives and nurses also emphasized that physicians could not be totally relieved from BFHI-related duties. Statistics as well as reports of success stories were used to convince physicians: “Once I had experienced some success on my own, I got committed to the program. From that time onwards, I worked according to the BFHI and I’ve to emphasize that others noticed my behavior as well” (Midwife 7). Moreover, a nurse unit manager commented that informing the manager of the unit about problems with physicians would help.

##### Tensions between care for mothers and care for babies

Participants debated the compatibility of the BFHI criteria with the provision of care oriented towards the needs of the mother. It was mainly midwives and gynecologists who were skeptical. Some of them had the feeling that BFHI, in particular the Ten Steps, focused too much on the baby and the needs of the mother were often neglected. A midwife expressed her concern as follows: “However, Baby-Friendly isn’t equal to mother-friendly” (Midwife 10). Others said that some colleagues supposed that meeting the BFHI criteria put pressure on mothers. Especially with respect to breastfeeding (Steps 5 and 8) and the ban on pacifiers (Step 9) there were multiple biases. A pediatrician mentioned: “[…] concepts such as ‘breastfeeding Taliban’ are circulating as well as ideas that infants aren’t allowed to use a pacifier, pacifiers are removed and mothers are forced to breastfeed” (Physician 10).

Although biases about the operation of BFHI requirements existed, interviewees indicated that the benefits of breastfeeding were beyond question. Yet, defining boundaries between promoting breastfeeding and starting to force mothers to breastfeed was considered a great, but solvable challenge. “We’ve the duty to implement it [BFHI] in a manner that women feel valued and their autonomy is guaranteed […]” (Physician 11).

By contrast to midwives and gynecologists, several pediatricians and some nurses felt that the BFHI was rather supportive to the care provided on maternity units. These interviewees felt that BFHI enabled staff to focus more precisely on babies’ needs, which was considered to be of primary importance. Pediatricians and nurses emphasized that babies were not able to vocalize their own needs, whereas mothers were able to do so: “The problem is that infants [as compared to mothers] have no voice. The baby can’t say it wishes breast-milk and wants as much skin-to-skin contact as possible with the mother” (Physician 10). One nurse unit manager also felt that BFHI criteria helped to improve care for the baby: “Looking at it from a baby’s perspective, multiple aspects become less absolute […] Babies can’t tell their moms ‘Stay here, don’t leave me’. Nobody would leave a two year old child alon.” (Nurse 5).

Overcoming these issues appeared to be quite difficult because this barrier largely refers to differences originating from employees’ professional education and orientation. However, again participants emphasized that referring to successful cases and discussing concerns and problems during team meetings helped to break down this barrier. Moreover, BFHI advocates indicated that optimal care for babies and their mothers are not in conflict.

##### Intra- and inter-professional discontinuation of the BFHI care-chain

Interviewees stressed that the BFHI care-chain was often interrupted within the same professional group during handing over of services or due to different working patterns of shifts. A pediatrician explained: “In particular, mostly at the end of the night when everybody is worn out and tired, giving formula is a lot easier than providing alternatives which are rather energy-consuming” (Physician 10).

Analysis also revealed that the BFHI care-chain is interrupted because of inter-professional discontinuation. Several participants were concerned that professional groups did not collaborate in an integrative or coordinated manner in terms of BFHI requirements. Especially anesthesiologists, as well as some pediatricians and gynecologists, asserted their right to work in accordance with their particular professional standards, which could differ from the Baby-Friendly procedures agreed upon in the hospital. Anesthesiologists often refused skin-to-skin contact after cesarean section because this constrained them in continuously monitoring the mother. Similarly, the manager of one maternity unit explained how pediatricians impeded continuous skin-to-skin contact after cesarean section: “In the beginning pediatricians felt that they first had to do vital checks on the baby and afterwards return the baby” (Physician 7). In another hospital, a pediatrician constantly determined the use of supplementary formula feeding by referring to pediatric guidelines. Yet, colleagues pointed out that, in many cases, the reasons for providing formula were not in accordance with the defined medical indication of BFHI.

Midwives and nurses emphasized that collaboration between their professional groups was less well-implemented and mothers were quickly handed over from the labor room to the maternity unit: “For example we pass the child over from the labor room to the unit only saying ‘breastfeeding yes/no’ […] yet specifying which positions have already been shown would make much more sense” (Midwife 10).

To improve the hand-over of mothers and babies, nurses and midwives said that sharing information had to be intensified between their professional groups and new ways of informing colleagues about the care already given had to be found. In one hospital, a seal for mothers’ records as well, as those of the babies, was introduced to document “informed primary weaning” (Physician 10) meaning that a woman has made a conscious decision not to breastfeed her baby. Another strategy how to overcome intra- and inter-professional discontinuation was additional documentation of e.g. reasons for formula feeding in the care record. As a consequence of the additional documentation, several interviewees hoped that other colleagues would be less *laissez faire* about formula feeding: “[…] I hope that formula feeding becomes more difficult because of documentation. I expect that colleagues will decide more consciously whether to give a bottle because they’ve to document reasons for it” (Midwife 10). Furthermore, a nurse explained that in her hospital they had temporarily introduced a documentation book to monitor skin-to-skin contact after cesarean section: “[…] We used a documentation form because we wanted to see what is happening and why mother and baby could be in bonding [skin-to-skin contact] or not” (Nurse 2). A midwife in another hospital commented similarly: “It’s the manager of midwives who sometimes does ‘inspection rounds’ on the unit. Similarly, she controls documentation forms in the records of mother and child. If she finds something suspicious, we’ll discuss it during the next team meeting. However, not every midwife is controlled every day” (Midwife 8).

##### Language and literacy barriers of mothers and their relatives

Language barriers as well as illiteracy among mothers repeatedly impaired the BFHI. Interviewees from all professional groups felt that educating mothers about breastfeeding (Step 3) was especially challenging: “The problem is, especially among women from Turkey, that they can’t read [German]. You give them an information folder, they smile at you and you think, great they’ve understood, but they just can’t read [German]” (Midwife 2).

As a response, maternity units had developed multilingual information materials, on the one hand, and, on the other hand, information was translated on the unit either by staff speaking the particular language or by relatives, often husbands: “For example, we’ve a colleague who is IBCLC-certified and her mother tongue is Turkish” (Midwife 4).

##### Expectations of mothers and their relatives

Another factor was expectations of mothers and their relatives. Interviewees from different professional groups often referred to Step 4 (skin-to-skin contact) of the Ten Steps. A pediatrician explained that skin-to-skin contact after natural birth was often interrupted because parents wanted to know the weight and length of their baby: “[…] coming back after 10 minutes you already find the baby on the scale because the mother wanted to know the birth weight” (Physician 6). Interviewees further explained that BFHI was impeded because some mothers brought their own formula. Several women, especially those with a different cultural background, believed that their babies would starve without formula. Some participants pointed out that, although from their professional perspective, giving formula to the baby was not necessary, it still happened. Interviewees said that trying to talk to the mother and to convince her not to give formula was a challenging endeavor: “[…] probably I can’t convince them [that formula is unnecessary] because they just don’t listen to me but rather listen to their own mothers or mothers-in-law. These are the ‘teachers’ in such cultures […]” (Nurse 2).

## Discussion

The aim of this study was to investigate health professionals’ perceptions of the different stages of BFHI implementation. Analysis revealed that in Austria multiple motives and prerequisites on maternity units themselves have to be present to become a Baby-Friendly hospital. Furthermore, the sub-categories under installation, as well as the facilitators of and numerous barriers to BFHI operation, have shown the complexity maternity units have to deal with.

Considering why organizations invest in health promotion, Pelikan provides two general reasons: (1) the decision can be orientated towards the purpose and function (purposive orientation) of a health promotion intervention for the organization or (2) towards the fact, that a health promotion intervention is in line with values and beliefs of the organization (value orientation) [29]. In relation to the BFHI, all participants mainly provided reasons that are part of a purposive orientation. BFHI as a marketing tool as well as a tool to improve existing services and collaboration go beyond the program’s underlying purpose of promoting and supporting breastfeeding. Although our participants emphasized that the advantages of breastfeeding for mothers and babies were beyond question, other, organizational purposes were pursued. Therewith, our findings expand on previous studies which highlight the importance of staff providing consistent information [17,18,21]. Moreover, following the theory on diffusion of innovations [30], to be one of those few BFHI-certified hospitals is a marketing feature as long as there are few BFHI-certified hospitals in Austria. Our findings further showed that to move from advocacy for the BFHI to the final decision, three prerequisites – individual persons in the role of change agents, consensus of managers of different professional groups, as well as consent of top management – had to be fulfilled within the hospital. The relevance of change agents in promoting change has also been considered within the broader literature on diffusion of innovations [30] and, in particular, within a review about diffusion of innovations in health service organizations [25]. Yet, our findings show that change agents were not purposively appointed by maternity units, but were already present on all three units and might be missing as a starting point in other non-BFHI-certified hospitals. These findings in combination with the fact that strong government endorsement of BFHI is absent in Austria might be one reason why, until now, less than one-fifth of Austrian hospitals have been designated Baby-Friendly. The experiences of Sweden [19] and New Zealand [24] have shown how national policy directives or external incentives can enhance BFHI-certification rates. In Austria, whether the recent roll-out actions for the dissemination of the BFHI, initiated at governmental level, will increase BFHI-certification rates remains to be seen.

The achievement of different steps or milestones before a program can be put into practice has been emphasized by logical models of program implementation in organizations [26,27]. Our study indicates that applying the principles of project management to initiate change was a matter of course for the three hospitals. The findings support the perception that project management, as part of quality management, has found its way into the hospital sector in Austria, not least the “quality commission” which has been mandated by law as a support unit in every Austrian hospital since 2004. Our findings emphasize the importance of audit and feedback to facilitate BFHI implementation, which are also highlighted within the broader literature on quality management in hospitals [31]. Furthermore, we conform to findings from an Australian study where a quality management approach was applied to facilitate BFHI implementation [32]. Installation activities, such as the manifestation of new work procedures in written form, resonate with previous research showing that written standards are widely used instruments in health care and are effective strategies to change the performance of health professionals [33]. Similarly, providing training to enhance professionals’ skills is a well-known activity to facilitate BFHI implementation [12,34,35].

Looking at the operation of the BFHI, our findings, like previous BFHI studies [17,18,20,21,36-38], emphasize the critical importance of readiness for change and organizational capacities including (1) skills of the staff, (2) management support, as well as (3) getting staff on board to facilitate BFHI. These findings might be a result of the fact that we have chosen BFHI-certified facilities and one facility preparing for BFHI-certification. Our study also supports findings from the broader health promotion literature, which identify organizational capacities as a basic prerequisite for successful implementation of health promotion interventions [39]. The barriers we found, largely relate to the Ten Steps rather than the Code or the mother-friendly care criteria. As mother-friendly care largely refers to practices before and during labor, as well as the fact that Austrian maternity units follow the principles of the “gentle birth” approach, postulated by Frédérick Leboyer and others, might explain why our data does not provide a lot of information on this aspect of the BFHI. With respect to the Code, all facilities emphasized that formula feeding was already constrained by a decision of the hospitals’ association. Our data shows rather that often either personal or professional attitudes of staff or of mothers and their relatives impeded BFHI. Similarly, Walsh et al. emphasize that staff’s personal views and understanding were often discordant with BFHI [23]. However, our findings expand on previous ones, in that they highlight differences in perceptions between different professional groups. Our findings show that the BFHI is not adopted equally by all professional groups and that physicians especially use their “professional autonomy” [40] by referring to their professional guidelines, to impede BFHI operation. Therefore, more attention should be paid to physician buy-in as well as the compatibility between professionals’ guidelines and BFHI guidelines.

Barriers related to employees’ personal and professional attitudes as well as mothers’ and relatives’ attitudes are particularly difficult to overcome because an organization cannot directly change these. Analysis revealed that structural support and additional resources accomplished during the installation stage fell short in facilitating change and additional activities would be required to overcome barriers resulting from individuals’ attitudes. Yet, as these attitudes can only be influenced indirectly, attempts to change individuals’ attitudes imply complex and continuing activities. In our settings, activities that were meant to overcome individuals’ attitudes and to facilitate BFHI as part of daily work routines as well as physician buy-in involved:

- **Additional monitoring and documentation:** Our data indicated that, in addition to the breastfeeding statistics that are required by the BFHI [4,13], our study sites monitored and documented other BFHI-related work, such as the duration of skin-to-skin contact after cesarean section or reasons for the provision of formula feeding. These additional activities enabled maternity units to visualize progress as well as existing problems. As a consequence, existing problems could be discussed and alternative solutions could be found. However, our participants also revealed that additional monitoring and documentation did not appear to be a sustainable activity to facilitate BFHI during daily work.
- **Process evaluation/self-evaluation:** Participants emphasized that it was not merely monitoring of outcomes that facilitated BFHI, but also the exchange with colleagues about putting BFHI into daily routines that was considered helpful. As with the additional monitoring and documentation, process evaluation facilitated self-reflection among employees and, as a consequence, supported them to find alternative strategies to solve particular problems.
- **Continuous commitment process:** Our findings suggest that besides individual readiness for change [17,18,20,21,37] as well as collective behavioral change [36,41], particular attention has to be paid to the fact that gaining commitment is an ongoing, iterative process already starting during the selection stage and being as relevant during the installation stage and operation stage. We expand on previous studies in that to gain commitment, information through e.g. kick-off, participation of staff in the project group and sub-groups as well as ongoing discussions during team meetings are needed to overcome barriers on the individual level and to create a so-called “critical mass”.
- **Differentiation and specialization:** To facilitate BFHI operation, participants emphasized that e.g. breastfeeding counseling should not be provided by every employee. Rather they suggested that employees with expert knowledge in breastfeeding counseling should be referred to by colleagues. Findings of training options offered showed that our hospitals trained a number of employees far beyond the official BFHI-requirements. In contrast to findings of Nickel et al. [36], it appeared that our hospitals had sufficient IBCLC-certified staff to provide breastfeeding counseling. In our study sites, intensive investment in differentiation and specialization of employees, such as the training towards IBCLC examination, helped to overcome barriers.
- **Structural changes of work procedures:** This aspect strongly relates to the previous one. Our participants noted that mere differentiation and specialization does not automatically translate into overcoming implementation barriers, as was also shown by Nickel et al. [36]. Our interviewees emphasized that the exclusive availability of e.g. IBCLC-certified staff was, to some extent, insufficient because these persons needed dedicated work time to apply their knowledge and expertise. Thus, our findings expand on previous studies because we have shown that to facilitate BFHI operation, duties on the unit have to be rearranged to fully utilize the advantages of trained staff.
- **Multilingual information and translation services:** Analysis has shown that also mothers and relatives – in particular their attitudes and expectations – can be a hindrance to successful BFHI operation. Additional information materials, which were translated into multiple languages, as well as verbal translation of information, facilitated the operation stage.

The strengths of this study are that multiple professional groups from different maternity units participated. The qualitative approach provided valuable insights into health professionals’ experiences and perceptions regarding the selection, installation and, ultimately, the operation of BFHI. However, the study settings were limited to one urban area of Austria and participation was voluntary. Thus, participants were only those who were interested in the study and who may have had more positive perceptions of the BFHI than non-participants.

## Conclusion

Current circumstances – without strong external support and incentives provided by the government – make a considerable increase in BFHI-certification rates unlikely to be realized. Our findings suggest that becoming a Baby-Friendly hospital requires a complex interplay of factors during the several stages of implementation. Moreover, hospitals seeking BFHI-certification may benefit from distinct and intensive investments in planning and preparation before BFHI is actually put into practice. Extensive information for staff but also for mothers and their relatives, continuous participation of health professionals as well as room for open debate before and during the operation stage may be basic activities to facilitate BFHI implementation in other hospitals. If these activities are continuously adjusted or even expanded during the operation of the BFHI, BFHI-certification could potentially increase and a sustainable BFHI implementation could be achieved.
